# Supplementary material for: Efficacy of nonviral gene transfer of human hepatocyte growth factor (HGF) against ischemic-reperfusion nerve injury in rats
Source: PLoS One. 2020 Aug 11;15(8):e0237156. doi: 10.1371/journal.pone.0237156 (PMC7418984; doi:10.1371/journal.pone.0237156)
Supplement: S1 Table — (DOCX) [file pone.0237156.s001.docx]

| **S1 Table. Primer sequences, TaqMan probe sequences, and thermocycle conditions used for RT-PCR** | | | | | | |
| --- | --- | --- | --- | --- | --- | --- |
| **mRNA** | **Sense primer**  **(5'-3')** | **Anti-sense primer (5'-3')** | **TaqMan probe**  **(5'-3')** | **Annealing temperature (°C)** | **Cycles** | **Size of PCR products (bp)** |
| P2X2 receptor | CCTCAAGCATTG CACATTTGA | TCGAGCCTCCGG AAAGAATA | CAGGACTCTGACCCA TACTGTCCCATCTTC | 60 | 40 | 201 |
| P2X3 receptor | CAGTGTTTCCCC TGGCTACA | GGTTGACGCAGT ACCCTTCA | TCATCCCCATTATCA GCTCGGT | 60 | 40 | 301 |
| P2X4 receptor | GGGTGAAGTTTT ATTCCAGC | GGGTGAAGTTTT CTGCAGCC | ACCAACATGATCGTC ACCGTGAACC | 60 | 40 | 301 |
| P2Y1 receptor | CTGCCTGCGGTC TACATCTT | CCACGCCACTGT ACCTGTGT | TGTTCATCATAGGCT TCCTTGGCAAA | 60 | 40 | 301 |
| TRPV1 | TTTTGGGAAGGG  TGACTCAGA | TGACAACAGAGCTGACGGTG | AGGCCTCTCCCATGG  ACTGCCC | 60 | 40 | 301 |
| GAPDH | CTTCACCACCAT GGAGAAGGC | GGCATGGACTGT GGTCATGAG | CCTGGCCAAGGTCAT CCATGACAACTTT | 60 | 40 | 238 |
| GAPDH, glyceraldehyde 3-phosphate dehydrogenase. TRPV1, transient receptor potential vanilloid receptor subtype 1 | | | | | | |
