# Supplementary material for: Efficacy of nonviral gene transfer of human hepatocyte growth factor (HGF) against ischemic-reperfusion nerve injury in rats
Source: PLoS One. 2020 Aug 11;15(8):e0237156. doi: 10.1371/journal.pone.0237156 (PMC7418984; doi:10.1371/journal.pone.0237156)
Supplement: S3 Table — (DOCX) [file pone.0237156.s003.docx]

S3 Table: Fascicular area and the density/number of endoneurial microvessels at the lower-thigh level of the right sciatic nerves at 3 weeks after ischemic-reperfusion injury

| Fascicular area (mm^2^) | | | | |
| --- | --- | --- | --- | --- |
|  | IRI+HGF | IRI | Control | Sham |
| R | 0.62 ± 0.06 | 0.64 ± 0.12 | 0.57 ± 0.11 | 0.58 ± 0.09 |
| L | 0.60 ± 0.04 | 0.55 ± 0.12 |  |  |

| Density of endoneurial microvessels (#/mm^2^) | | | | |
| --- | --- | --- | --- | --- |
|  | IRI+HGF | IRI | Control | Sham |
| R | 88 ± 10* | 66 ± 7 | 66 ± 6 | 69 ± 10 |
| L | 90 ± 11* | 63 ± 9 |  |  |

**p*<0.01: IRI+HGF *vs.* IRI, control & sham

| Total number of endoneurial microvessels (#/nerve) | | | | |
| --- | --- | --- | --- | --- |
|  | IRI+HGF | IRI | Control | Sham |
| R | 54 ± 5* | 42 ± 6 | 39 ± 9 | 40 ± 7 |
| L | 54 ± 8* | 34 ± 7 |  |  |

**p*<0.05: IRI+HGF *vs*. IRI, control & sham
