# Supplementary material for: Efficacy of nonviral gene transfer of human hepatocyte growth factor (HGF) against ischemic-reperfusion nerve injury in rats
Source: PLoS One. 2020 Aug 11;15(8):e0237156. doi: 10.1371/journal.pone.0237156 (PMC7418984; doi:10.1371/journal.pone.0237156)
Supplement: S4 Table — (DOCX) [file pone.0237156.s004.docx]

S4 Table: Fascicular area and the density of endoneurial microvessels at the lower-calf level of the right tibial nerves at 3 weeks after ischemic-reperfusion injury

| Fascicular area (mm^2^) | | | |
| --- | --- | --- | --- |
|  | IRI+HGF | IRI | Control |
| R | 0.17 ± 0.03 | 0.17 ± 0.03 | 0.16 ± 0.02 |
| L | 0.60 ± 0.04 | 0.55 ± 0.12 |  |

| Density of endoneurial microvessels (#/mm^2^) | | | |
| --- | --- | --- | --- |
|  | IRI+HGF | IRI | Control |
| R | 111 ± 17* | 103 ± 15 | 88 ± 17 |
| L | 113 ± 19* | 89 ± 13 |  |

**p*<0.05: IRI+HGF *vs.* IRI & control
