# Supplementary figures and images for: Efficacy of nonviral gene transfer of human hepatocyte growth factor (HGF) against ischemic-reperfusion nerve injury in rats
Source: PLoS One. 2020 Aug 11;15(8):e0237156. doi: 10.1371/journal.pone.0237156 (PMC7418984; doi:10.1371/journal.pone.0237156)

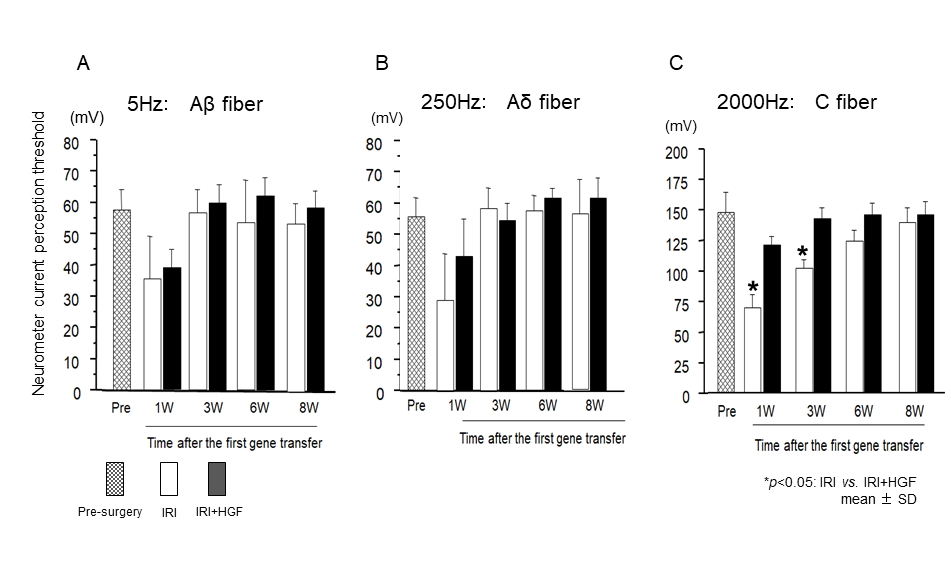

Supplement: S1 Fig — Neurometer measurements of stimulus threshold at 5 (A), 250 (B) and 2,000 (C) Hz to the plantar surface of the left hind paw in IRI (open bars) and IRI+HGF (solid bars) groups, and pre-surgical levels (Pre OP, checkered bars). Data are expressed as mean ± SD (n = 6 for each group at each time point). *p < 0.01: IRI vs. IRI+HGF. (TIF) [file pone.0237156.s005.tif]

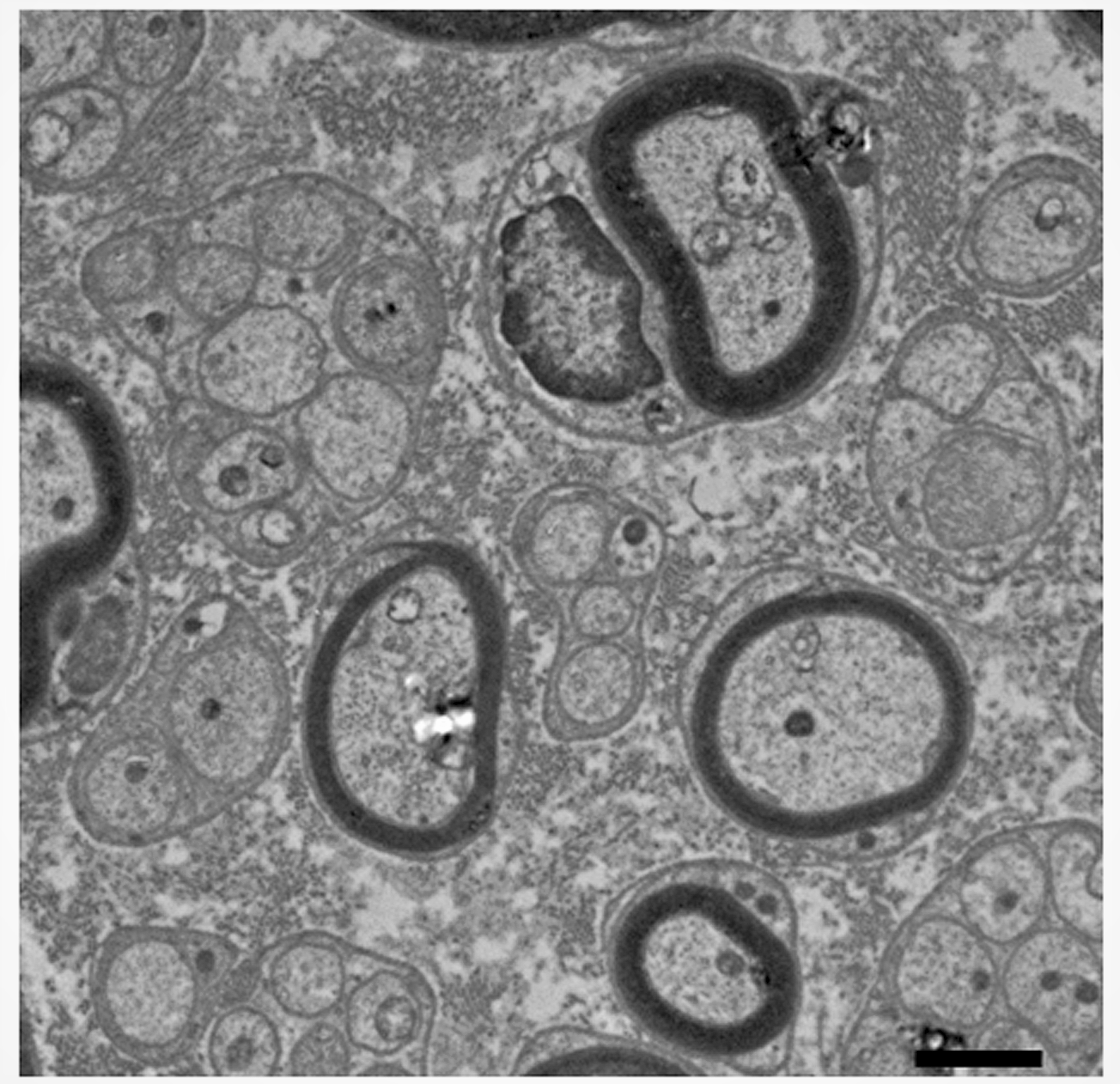

Supplement: S2 Fig — Note myelinated nerve fibers with disproportionally thin myelin relative to axon. area Bar = 2μm. (TIF) [file pone.0237156.s006.tif]

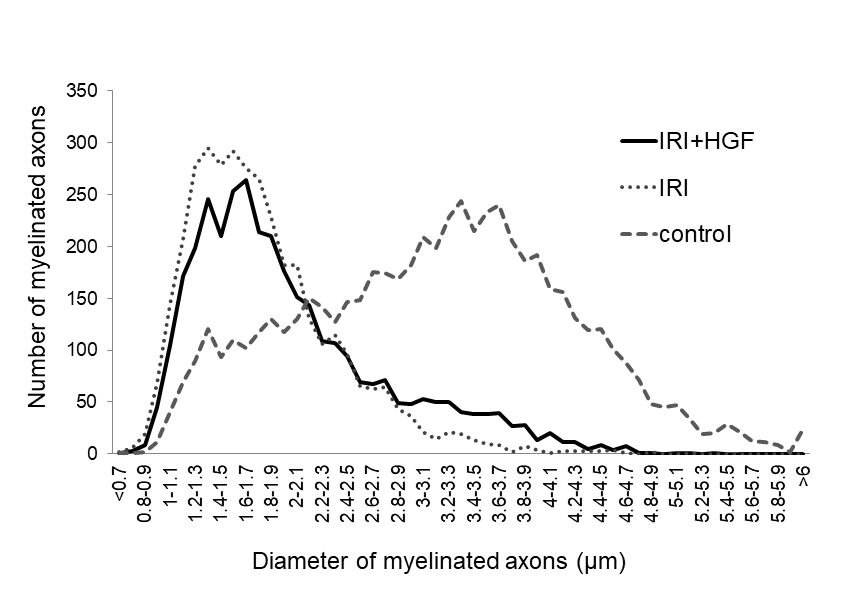

Supplement: S3 Fig — (TIF) [file pone.0237156.s007.tif]

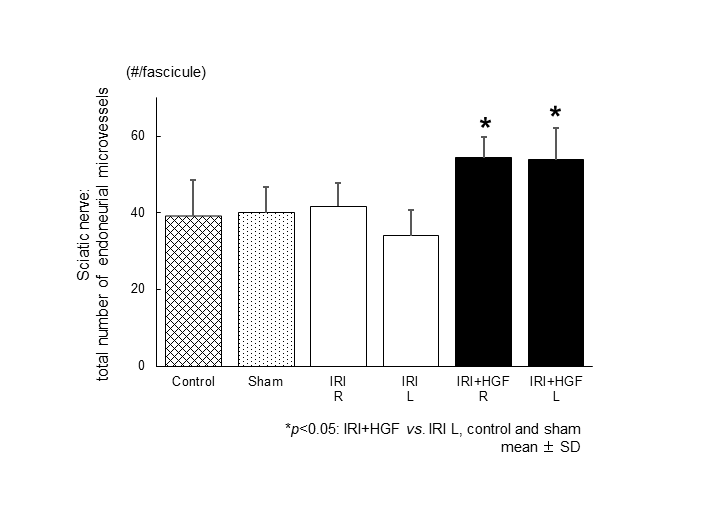

Supplement: S4 Fig — *p < 0.01: IRI+HGF vs. IRI. (TIF) [file pone.0237156.s008.tif]

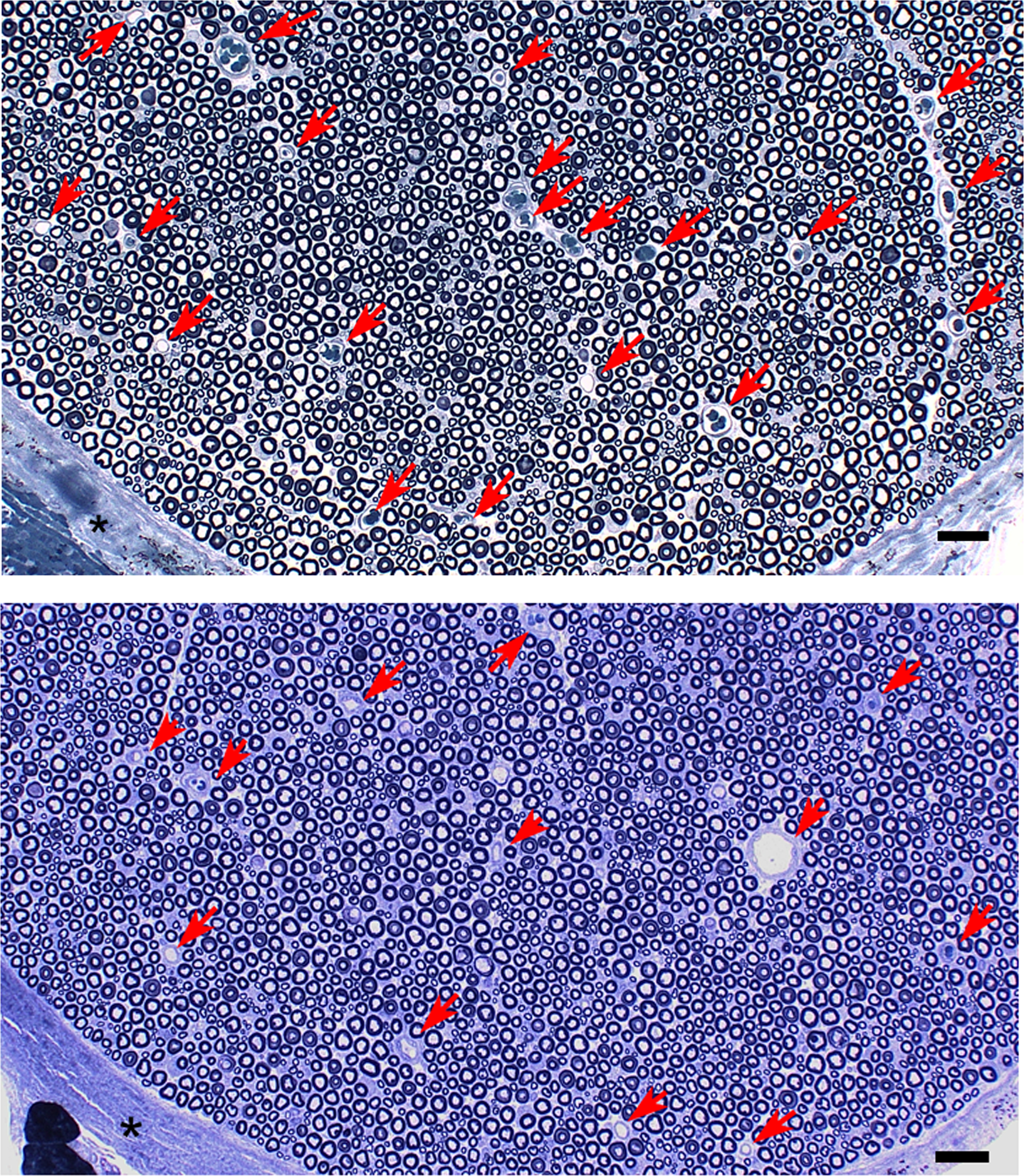

Supplement: S5 Fig — Semi-thin transverse sections at the upper thigh level of the contralateral (left) sciatic nerves at 3 weeks post-surgery showing endoneurial microvessels (arrows) in IRI+HGF (A) and IRI (B) groups. The number of endoneurial microvessels in HGF-treated nerve (A) was apparently greater than in IRI nerve (B). Bars = 25μm. *perineurium. (TIF) [file pone.0237156.s009.tif]

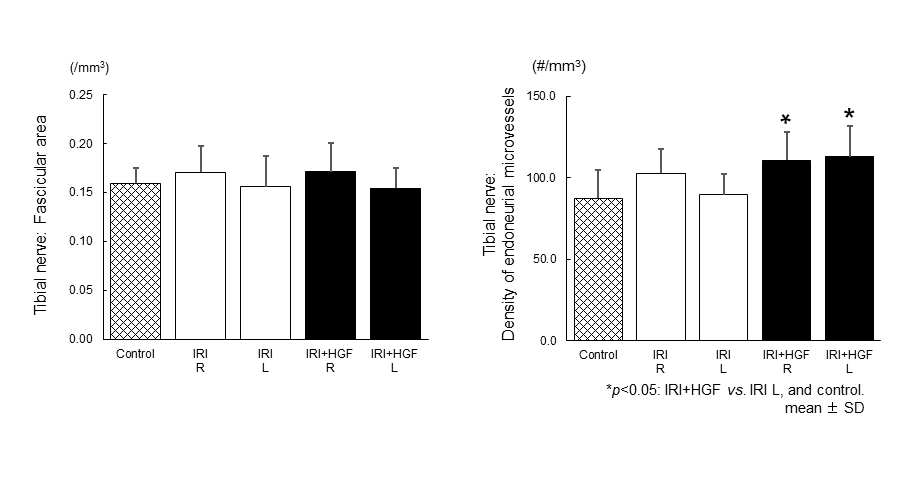

Supplement: S6 Fig — (TIF) [file pone.0237156.s010.tif]

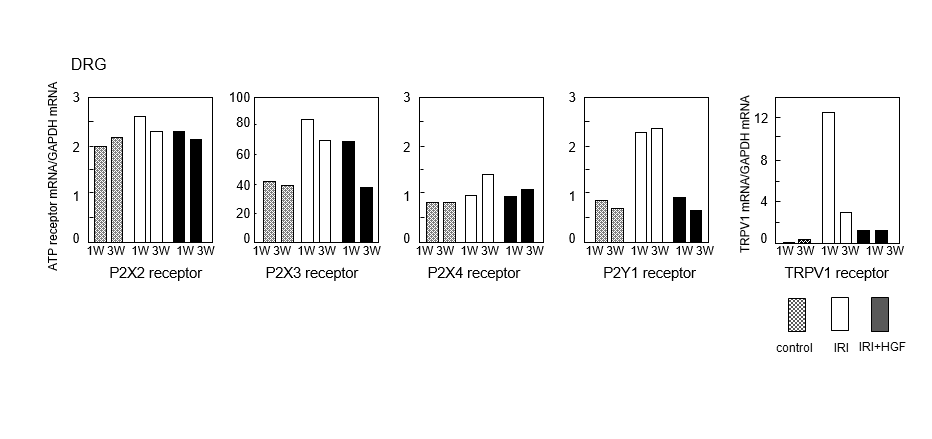

Supplement: S7 Fig — Data are expressed as mean ± SD (n = 6 for each group at each time point). (TIF) [file pone.0237156.s011.tif]
