# Supplementary material for: Efficacy of nonviral gene transfer of human hepatocyte growth factor (HGF) against ischemic-reperfusion nerve injury in rats
Source: PLoS One. 2020 Aug 11;15(8):e0237156. doi: 10.1371/journal.pone.0237156 (PMC7418984; doi:10.1371/journal.pone.0237156)
Supplement: Supplementary file 5 [file pone.0237156.s002.docx]

		Pre		Day 1		Day 2		Day 3		Day 4		Week 1		Day 10		Week 2		Day 17		Week 3		Day 24		Week 4		Week 5		Week 6		Week 7		Week 8	
		R	L	R	L	R	L	R	L	R	L	R	L	R	L	R	L	R	L	R	L	R	L	R	L	R	L	R	L	R	L	R	L
Sham	1	15	15	15	15	15	15	15	15	15	15	15	15	15	15	15	15	15	15	15	15	15	15	26	26	10	15	15	26	15	26	15	15
	2	26	26	26	15	15	26	26	15	26	26	26	26	26	15	26	26	26	26	26	26	26	26	15	26	15	26	15	15	26	26	26	26
	3	15	15	15	15	26	15	15	15	15	15	15	15	15	15	15	15	15	15	15	15	15	15	15	15	15	15	15	15	15	15	15	15
	4	15	15	10	10	15	15	15	26	15	15	15	15	15	26	15	15	15	26	15	15	15	15	15	15	26	26	26	26	15	15	15	15
	5	15	15	15	15	15	15	15	15	15	15	10	15	15	15	15	15	15	15	15	15	15	15	10	15	15	15	15	15	15	15	15	15
	6	15	15	15	15	15	15	15	15	15	15	15	15	15	15	15	15	15	15	15	15	15	15	15	15	10	15	15	15	15	15	15	15
	mean	17	17	16	14	17	17	17	17	17	17	16	17	17	17	17	17	17	19	17	17	17	17	16	19	15	19	17	19	17	19	17	17
	SD	4	4	5	2	4	4	4	4	4	4	5	4	4	4	4	4	4	6	4	4	4	4	5	6	6	6	4	6	4	6	4	4
Control	1	15	15	15	15	15	15	15	15	15	15	15	15	15	15	15	15	15	15	15	15	15	15	15	15	15	15	15	15	15	26	26	26
	2	15	15	15	15	15	15	15	15	15	15	15	26	15	15	15	15	26	26	26	26	15	26	26	15	26	26	26	26	15	26	15	15
	3	15	15	15	15	26	26	15	15	15	15	10	15	10	15	15	15	15	15	15	15	15	15	15	15	15	15	15	15	15	15	15	15
	4	15	15	15	26	15	15	15	15	26	15	15	15	15	15	26	15	15	15	15	15	10	15	10	15	10	15	10	15	10	15	10	15
	5	26	26	26	26	15	15	26	26	26	26	26	15	26	15	26	15	15	26	26	15	26	15	15	15	15	15	15	15	26	15	15	15
	6	15	15	15	15	15	15	15	15	15	15	15	26	15	15	15	15	15	15	15	15	15	15	15	15	15	15	15	15	15	15	15	15
	mean	17	17	17	19	17	17	17	17	19	17	16	19	16	15	19	15	17	19	19	17	16	17	16	15	16	17	16	17	16	19	16	17
	SD	4	4	4	6	4	4	4	4	6	4	5	6	5	0	6	0	4	6	6	4	5	4	5	0	5	4	5	4	5	6	5	4
IRI+HGF	1	15	15	1.4	8	1.4	10	1.4	10	2	15	2	15	4	26	2	10	4	26	4	26	6	15	10	26	10	15	15	26	15	26	15	15
	2	15	26	1.4	6	1.4	10	2	10	1.4	10	2	10	4	15	4	26	6	15	15	15	15	15	15	26	15	26	15	15	26	26	26	26
	3	15	15	1.4	10	2	10	2	15	1.4	15	6	15	4	26	8	15	10	26	15	15	15	15	15	15	15	15	15	15	15	15	26	15
	4	15	15	1.4	6	2	6	2	10	2	15	4	15	6	15	8	15	8	15	10	26	10	26	10	15	26	26	26	26	15	15	15	15
	5	15	15	2	10	1.4	10	1.4	10	1.4	10	2	15	2	10	6	15	8	10	10	15	10	15	10	15	15	15	15	15	15	15	15	15
	6	15	15	1.4	10	1.4	6	2	10	2	10	1.4	15	4	15	4	15	4	15	8	15	10	15	15	15	10	15	15	15	15	15	15	15
	mean	15	17	2	8	2	9	2	11	2	13	3	14	4	18	5	16	7	18	10	19	11	17	13	19	15	19	17	19	17	19	19	17
	SD	0	4	0	2	0	2	0	2	0	3	2	2	1	7	2	5	2	7	4	6	3	4	3	6	6	6	4	6	4	6	6	4
IRI	1	15	15	1.4	8	1.4	6	1.4	8	1.4	10	1.4	10	1	15	2	15	4	15	4	15	4	15	4	15	8	15	10	15	10	26	26	26
	2	15	15	1.4	8	1.4	10	1.4	10	1.4	15	1.4	10	1.4	15	1.4	26	1.4	26	2	26	2	26	4	26	6	26	8	26	15	26	15	15
	3	26	15	2	10	2	10	2	10	2	10	1.4	8	2	10	2	10	2	10	2	15	2	15	2	15	4	26	6	15	10	15	15	15
	4	15	15	2	10	1.4	6	1.4	8	2	15	2	15	1.4	15	2	15	2	15	2	10	2	10	2	15	6	15	6	15	10	15	10	15
	5	15	26	2	10	2	8	1.4	10	1.4	10	1.4	15	1.4	15	2	15	2	15	4	15	2	15	4	15	8	15	8	15	15	15	15	15
	6	15	15	1.4	10	1.4	10	1.4	10	1.4	10	1.4	10	1.4	10	1.4	15	2	15	2	15	2	15	2	15	6	15	6	15	10	15	15	15
	mean	17	17	2	9	2	8	2	9	2	12	2	11	1	13	2	16	2	16	3	16	2	16	3	17	6	19	7	17	12	19	16	17
	SD	4	4	0	1	0	2	0	1	0	3	0	3	0	3	0	5	1	5	1	5	1	5	1	4	2	6	2	4	3	6	5	4
